# Human predation contributed to the extinction of the Australian megafaunal bird *Genyornis newtoni* ∼47 ka

**DOI:** 10.1038/ncomms10496

**Published:** 2016-01-29

**Authors:** Gifford Miller, John Magee, Mike Smith, Nigel Spooner, Alexander Baynes, Scott Lehman, Marilyn Fogel, Harvey Johnston, Doug Williams, Peter Clark, Christopher Florian, Richard Holst, Stephen DeVogel

**Affiliations:** 1 Institute of Arctic and Alpine Research, University of Colorado, Boulder, Colorado 80309-0450, USA; 2 Department of Geological Sciences, University of Colorado, Boulder, Colorado 80309-0399, USA; 3 Department of Environment and Agriculture, Curtin University, Perth, Western Australia 6102, Australia; 4 Research School Earth Sciences, Australian National University, Canberra, Australian Capital Territory 2601, Australia; 5 National Museum Australia, GPO Box 1901, Canberra, Australian Capital Territory 2601, Australia; 6 Institute for Photonics and Advanced Sensing and School of Physical Sciences, University of Adelaide, Adelaide,, South Australia 5005, Australia; 7 Defence Science and Technology Group, Edinburgh, South Australia 5111, Australia; 8 Western Australian Museum, Locked Bag 49, Welshpool DC, Western Australia 6986, Australia; 9 School of Natural Sciences, University of California, Merced, 95343 California, USA; 10 Office Environment and Heritage, Box 318, Buronga, New South Wales 2739, Australia; 11 Access Archaeology & Heritage, Box 816, Moruya, New South Wales 2537, Australia; 12 Infrastructure Planning and Natural Resources, Box 363, Buronga, New South Wales 2739, Australia; 13 289 Churchill Avenue, Subiaco, Western Australia 6008, Australia

## Abstract

Although the temporal overlap between human dispersal across Australia and the disappearance of its largest animals is well established, the lack of unambiguous evidence for human–megafauna interactions has led some to question a human role in megafaunal extinction. Here we show that diagnostic burn patterns on eggshell fragments of the megafaunal bird *Genyornis newtoni*, found at >200 sites across Australia, were created by humans discarding eggshell in and around transient fires, presumably made to cook the eggs. Dating by three methods restricts their occurrence to between 53.9 and 43.4 ka, and likely before 47 ka. *Dromaius* (emu) eggshell occur frequently in deposits from >100 ka to present; burnt *Dromaius* eggshell first appear in deposits the same age as those with burnt *Genyornis* eggshell, and then continually to modern time. Harvesting of their eggs by humans would have decreased *Genyornis* reproductive success, contributing to the bird's extinction by ∼47 ka.

## Abstract (web summary)

The impact of humans on megafaunal extinction is Australia is unclear. Here, the authors show burn patterns on eggshells of the extinct megafaunal bird, *Genyornis newtoni*, created by humans across Australia, suggesting that human predation contributed to the extinction of this bird around 47 thousand years ago.

## Graphical abstract

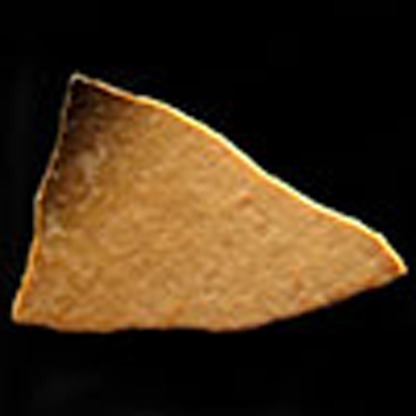

More than 170 years after the discovery of skeletal remains of giant vertebrates preserved in Australian caves^1^, and later in dry lakebeds^2^, the cause of their demise is still debated. Most scientific studies are directed at evaluating the two dominant explanations for extinction: human impact or climate change^3^. As dating methods have improved, chronologies for human dispersal across Australia and for the last surviving megafauna have become more accurate and more precise^4^,^5^,^6^. Although the date of initial human arrival on mainland Australia remains uncertain, populations were established over most of the continent by at least 47 ka (refs 6, 7). Although many elements of the Australian megafauna (those animals >45 kg body mass) lack firm extinction timelines, last appearance dates for taxa that occur most frequently in the fossil record are between 50 and 40 ka (refs 4, 5, 8), indicating a temporal overlap between humans and megafauna^9^. The climate of Australia was drying gradually between 60 and 40 ka, but neither the rate nor magnitude of change were more severe than during earlier Pleistocene climate shifts^10^,^11^. The lack of evidence for unprecedented climate change between 60 and 40 ka and survival of megafauna during earlier more extreme climate fluctuations^12^ implies that climate change is unlikely to be the sole cause of megafaunal extinction, leaving human agency more likely to have been the decisive factor, with modest additional stress from increasing aridity potentially a contributing factor^13^,^14^. Human colonizers may stress megafaunal populations by predation or by altering habitats so that dietary resources are reduced, with recent studies emphasizing the impacts of even modest hunting, especially for long-lived large vertebrates with low reproductive rates^15^,^16^. Although the argument for human predation contributing to widespread extinction in Australia is on firm grounds from ecological theory, little supporting evidence is available in the form of kill sites or unambiguous human modification of skeletal remains despite 50 years of systematic archaeological fieldwork. This lack of evidence has been used to argue against human predation as a cause of megafaunal extinction^17^. The absence of kill sites in Australia compared with the Americas has been postulated to be a consequence of the much earlier date for Australian extinctions, diminishing the probability of their preservation^4^,^18^,^19^. Here we provide direct evidence from sites across most of the continent that humans preyed on at least one element of the Australian megafauna, harvesting eggs of *Genyornis newtoni*, an extinct, 200 kg flightless bird, and leaving diagnostic burnt eggshell fragments as evidence of their activities. In the largest series of dates collated for any Australian megafaunal species, we demonstrate that burnt *Genyornis* eggshell only occur during a window between 53.9 and 43.4 ka, and likely before 47 ka, documenting megafaunal predation by humans as they dispersed across the continent.

## Results

### *Genyornis* eggshell

**Figure 1:**
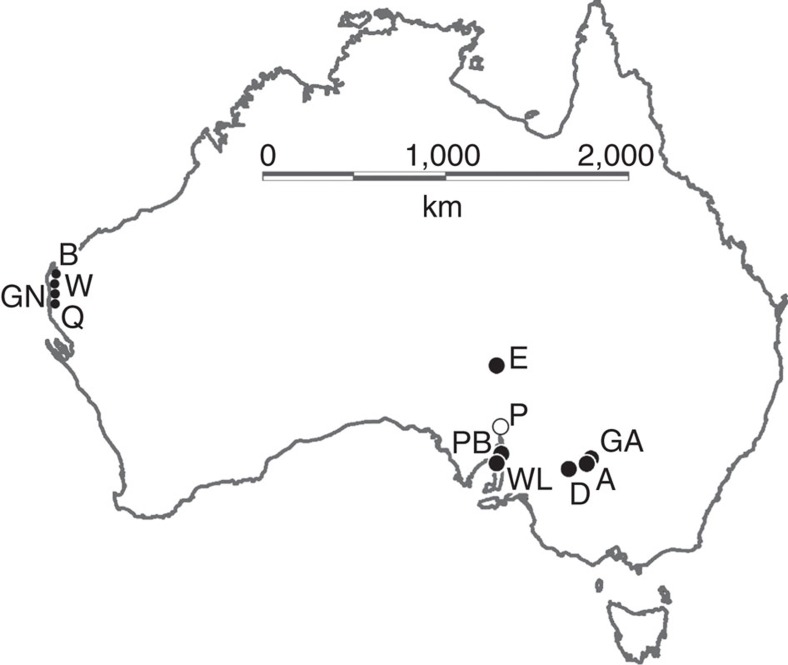
Regions across Australia from which diagnostic burnt *Genyornis* eggshell were collected. Map of Australia showing the ten regions from which burnt *Genyornis* eggshell fragments have been collected (solid circles) and one other region where other key samples were collected (open circle). A: Arumpo Station, NSW; GA: Garnpung Station, NSW; D: Lower Darling River, NSW; WL: Wallaroo, SA; PB: Port Broughton, SA; P: Port Augusta region, SA; E: Lake Eyre, SA; Q: Sites on and around Quobba Station, WA; GN: Sites on and around Gnaraloo Station, WA; W: Sites on and around Warroora Station, WA; B: Bullara, Ningaloo, and Cardabia stations, WA.

Fragments of *Genyornis* eggshell are found in recently deflated sand dunes where the birds nested, with morphological features differentiating *Genyornis* eggshell from those of *Dromaius* (emu)^20^. We analysed *Genyornis* eggshell from nearly 2,000 localities across Australia; none is clearly younger than 50±5 ka, whereas *Dromaius* eggshell are commonly found in the same regions from >100 ka to the present^8^. Field studies in ten regions across the continent (Fig. 1) yielded >200 collections that contain variably blackened *Genyornis* eggshell, frequently blackened at only one end of the fragment, suggestive of irregular heating patterns.

### Diagnostic patterned burning

**Figure 2:**
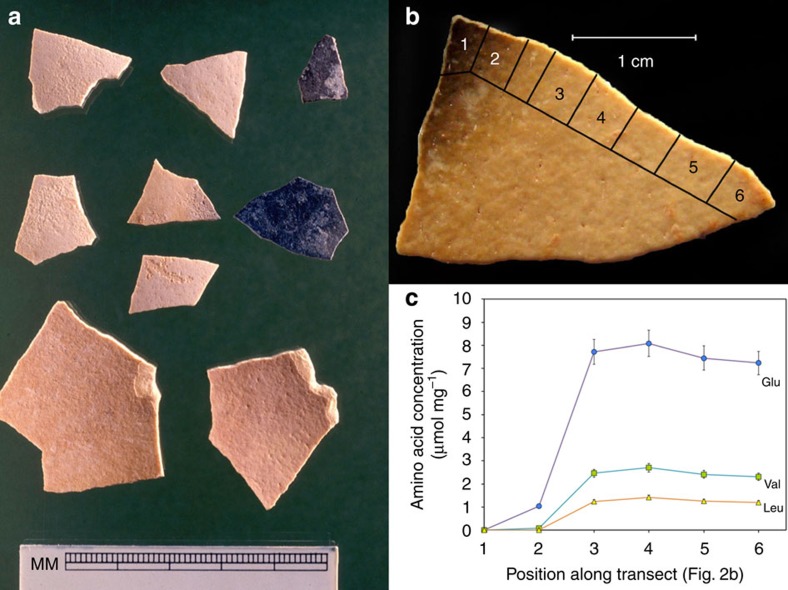
Characterizing blackened *Genyornis* eggshell. (**a**) Excavated variably burnt and unusually broken *Genyornis* eggshell from the Wood Point site, Port Broughton, Spencer Gulf, SA (PB, Fig. 1). (**b**) *Genyornis* eggshell fragment from region W (Fig. 1), blackened only at one end, with locations of samples for amino-acid analysis (**c**). (**c**) Concentrations of the stable amino acids glutamic acid (Glu), valine (Val) and leucine (Leu) are reduced in direct proportion to the degree of visual blackening, with samples 3–6 exhibiting only slightly lower concentrations than in unheated fragments of the same egg; ±1σ uncertainties (7%) based on duplicate analyses. Unheated fragments of the same egg exhibit 10% inter-eggshell variability for the same amino acids (6 fragments analysed). Less stable amino acids show similar patterns. Transects through other similarly blackened as well as fully blackened fragments are given in Supplementary Figs 1 and 2.

To test whether blackened eggshell is diagnostic of high temperatures, we sub-sampled partially blackened fragments for amino-acid analysis. We found that the blackened ends had been heated sufficiently to decompose all amino acids, with decreasing decomposition away from the burnt end, eventually reaching amino-acid concentrations similar to those in unburnt fragments (Fig. 2). Such a strong decomposition gradient can only be accomplished if the blackened end was briefly in contact with a localized high-heat-source, likely an ember; rapid amino-acid decomposition requires temperatures ≥500 °C (ref. 21). Graded amino-acid decomposition is apparent in transects across other variably blackened fragments, whereas fully blackened eggshell are devoid of amino acids, consistent with sustained high heat over the entire fragment (Supplementary Figs 1 and 2).

In most collections with burnt eggshell, we found examples of partially and wholly burnt fragments, some burnt so severely that their natural curvature was flattened, and occasionally reversed, as well as many visually unburnt fragments. These collections often form a tight cluster <3 m in diameter without other eggshell nearby, suggesting the source was a single, or small number of eggs. We conclude that natural wildfire could not produce such steep thermal gradients within and between nearby eggshell fragments, as it requires an untenable combination of circumstances. Rather, these characteristics are most consistent with humans harvesting one or more eggs from a nest, making a fire and presumably cooking the egg. For the same reason that it is possible to boil water in a paper cup over a fire without burning the cup, cooking an egg in a manner that does not cause the egg to explode, will not char the eggshell. Records of traditional Aboriginal cooking of emu eggs describe a relatively slow cooking of the eggs, either wrapped in vegetation or in hot ashes in a hole dug in the ground for that purpose, from which the egg would be removed and rotated or shaken frequently, then repositioned^22^. After cooking, we presume that eggshell fragments were discarded randomly in and around the fire. We find similarly burnt *Dromaius* eggshell in unambiguous late Holocene archaeological contexts along with other burnt food debris (Supplementary Fig. 3), and burnt ostrich eggshell is found in similar archaeological settings in Africa^23^,^24^.

Burnt *Genyornis* eggshell fragments are most common in coastal sand dunes of Western Australia (WA). Of 567 collections from four WA regions that contained *Genyornis* eggshell, 192 included burnt fragments, with much smaller proportions in the Darling (1 of 189 collections) and around Lake Eyre, the driest sector of the continent (1 of 542 collections; Supplementary Data 1). Although the Willandra (A, GA) and Spencer Gulf (WL, PB) regions each had few collections, burnt fragments occurred relatively frequently (2 of 6 and 10 of 23, respectively; Supplementary Fig. 5).

### Dating burnt eggshell

**Figure 3:**
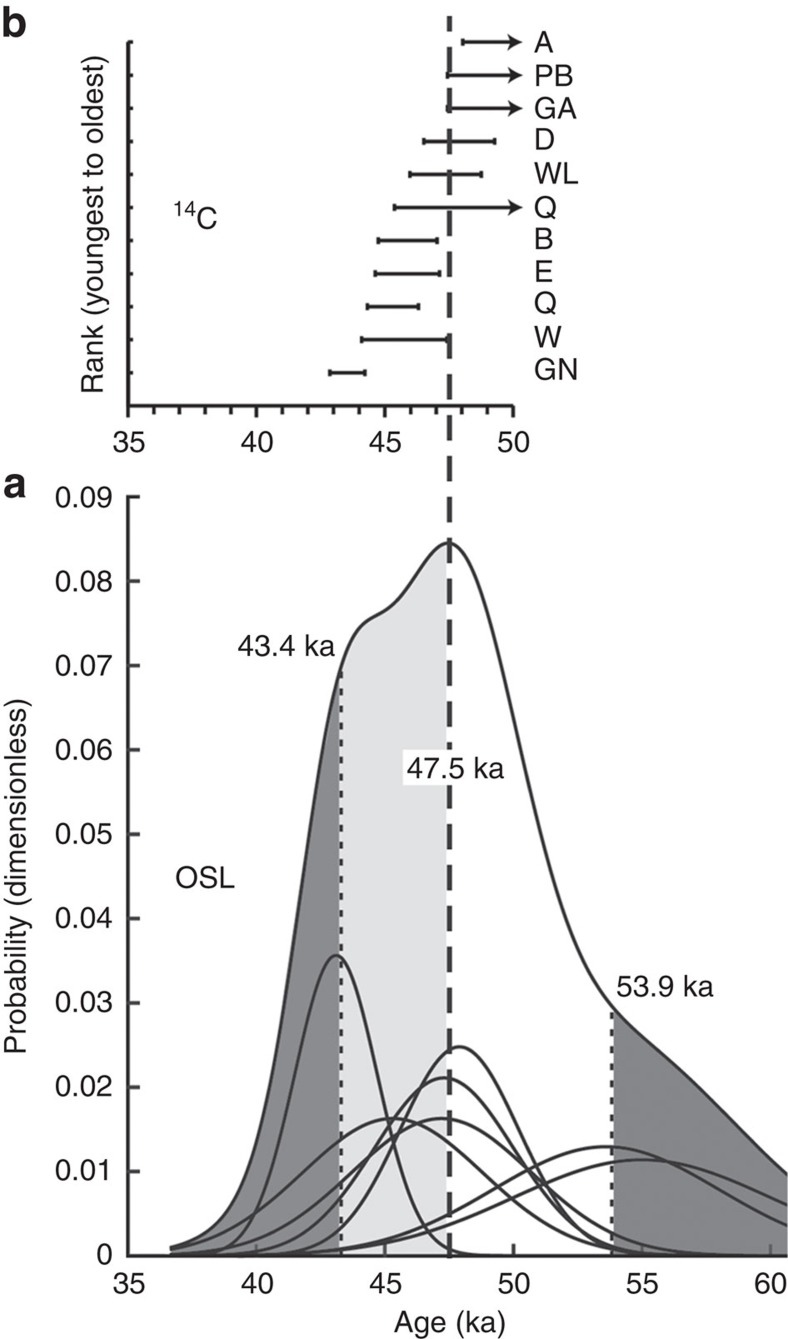
Luminescence and radiocarbon dating of burnt *Genyornis* eggshell. (**a**) Cumulative probability age distribution derived by summing the individual OSL Gaussian probability distributions for seven sites with burnt *Genyornis* eggshell (Table 1). The median age is 47.5 ka, with the light dashed lines representing the 16th and 84th percentiles of the aggregate distribution, similar to ±1σ in a Gaussian distribution. (**b**) Calibrated age ranges (±1σ) for 11 ^14^C dates on unburnt *Genyornis* eggshell associated with burnt fragments (Table 1), omitting the two dates from Warroora (W, Fig. 1) that are significantly younger than OSL dates from the same sites (no overlap at ±1σ; Table 1). Vertical heavy dashed line represents the age that most closely satisfies the ^14^C dates (with one exception), suggesting that burnt *Genyornis* eggshell is unlikely to be younger than 47.5 ka. Unshaded region in **a** represents the most likely age range for burnt *Genyornis* eggshell, although a range including the lightly shaded region cannot be confidently excluded. ^14^C dates are plotted with increasing minimum age upward; letters refer to regions located on Fig. 1.

To evaluate whether burnt *Genyornis* eggshell only coincide with a human presence in the landscape, we obtained absolute and relative dates using optically stimulated luminescence (OSL), radiocarbon (^14^C) and amino-acid racemization (AAR). Seven sites with burnt *Genyornis* eggshell in a stratigraphic context and eight other stratified sites with unburnt *Genyornis* eggshell have been dated by OSL (Table 1). The cumulative sum of the individual OSL ages and their uncertainties for collections with burnt eggshell yield an aggregate median age of 47.5 ka and a range from 53.9 to 43.4 ka (Fig. 3a), where the upper and lower bounds correspond to the 16th and 84th percentiles of the aggregate distribution (roughly equivalent to ±1σ in a normal distribution). Eight other collections with *Genyornis* eggshell that lack burnt fragments, but for which AAR indicates they are among the youngest in each region, have a median OSL age of 51.5±4.9 ka (Table 1).

*Genyornis* eggshell were dated directly by ^14^C using accelerator mass spectrometry following rigorous pretreatment. However, as in other carbonate media from terrestrial settings, eggshell calcite is subject to slow diffusion of younger carbon from its surroundings. For samples older than 40 ka, small amounts of young carbon will result in apparent ages significantly younger than their true age^25^,^26^. For example, we obtained finite ^14^C ages <46 ka on *Genyornis* eggshell from two collections dated ≥70 ka by OSL and/or AAR (Table 1), despite >50 ka background dates in standards. This demonstration of exchange with younger carbon suggests that all ^14^C dates >40 ka should be regarded as minimum ages. Unburnt *Genyornis* eggshell from 13 collections with burnt fragments, including all regions in Fig. 1, were dated by ^14^C (Table 1). Calibrated ^14^C ages for two of the Warroora sites are significantly younger than their corresponding OSL ages (no overlap at ±1σ; Table 1) and are considered anomalously young as a result of carbon exchange. For the remaining 11 samples, all but 1 have minimum calibrated ^14^C ages ≥44 ka. Considered collectively, calibrated ±1σ age ranges for the nine finite and two non-finite ages constrain a likely minimum calendar age for burnt *Genyornis* to 47.5 ka (Fig. 3b), although somewhat younger ages cannot be conclusively ruled out. These dates refine earlier estimates for *Genyornis* extinction of 50±5 ka (ref. 8) to 47.5±2.5 ka.

**Figure 4:**
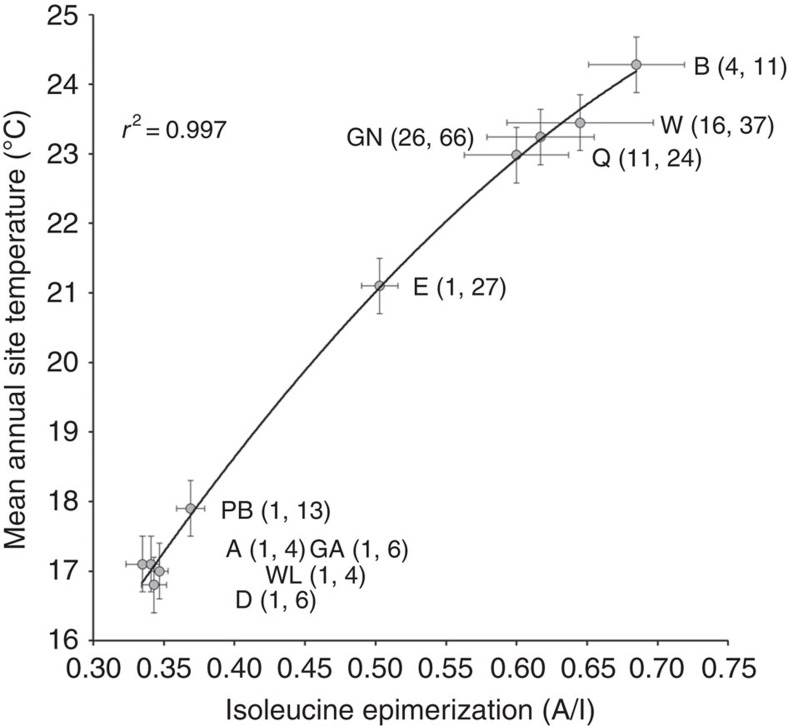
Extent of isoleucine epimerization in *Genyornis* eggshell compared to their current regional temperatures. Average A/I in *Genyornis* eggshell from 63 collections with burnt fragments recovered from all ten regions (Fig. 1) for which a secure A/I could be determined, plotted against their current (1960-1990 AD) mean annual temperature (MAT), each with its ±1σ uncertainty (Supplementary Data 2). Letters refer to regions in Fig. 1, numbers in parenthesis are the number of collections from each region, and the number of different eggshell fragments analysed in each region that contribute to the mean A/I value. The close fit to a simple polynomial regression with a trend towards ever-higher A/I for higher regional MATs, an expected result due to the exponential dependency of racemization rate on temperature, is consistent with, although not in itself proof of, all samples being of the same age. Age differences in excess of 5 ka in almost all cases would result in a significantly poorer polynomial fit.

Eggshell relative age is constrained by AAR in intracrystalline protein residues^27^ isolated from physically and chemically cleaned *Genyornis* eggshell (*n*=3,877)^28^. The amino acid isoleucine epimerizes to its non-protein diastereomer alloisoleucine at a rate dependent on temperature, with their ratio (A/I) reflecting time and the effective diagenetic temperature for each sample. For collections buried ≥2 m and not subjected to any other heat source, effective diagenetic temperature is set by the integrated mean annual temperature (MAT) since the egg was laid^28^. However, the extra energy imparted to fire-heated eggshell accelerates racemization. To minimize this effect, we select only visually unburnt fragments for AAR analysis, and we analyse multiple fragments from each collection containing burnt fragments. Yet, even visually unburnt fragments often exhibit variably high A/I values, presumably because they were heated at temperatures sufficient to accelerate racemization but too low to create black carbon. Cooking temperatures ≤100 °C for <1 h will not blacken eggshell or significantly raise A/I, whereas brief transient heating >150 °C but well below 500 °C accelerates racemization without blackening the eggshell. To prevent unblackened but fire-heated samples from biasing our interpretations, we screen our results, limiting summary plots to those collections for which the two (or more) lowest A/I differ by no more than 0.02 A/I units, thereby excluding heated (higher) A/I values; for a few collections, we used the lowest A/I if that was the only analysis within 1 σ of the region's mean A/I. We measured A/I in over 550 *Genyornis* eggshell fragments from 84 collections that also contain burnt fragments. In all, 63 of the 84 collections met our screening criteria, from which we compute regionally averaged A/I (Supplementary Data 2), and compare those to their corresponding regional MAT in Fig. 4. The close approximation to a simple second-order polynomial regression (*r*^2^=0.99) is consistent with the exponential dependence of racemization rate on temperature, based on kinetics derived in ref. 28, and the high correlation coefficient is consistent with a similar age for all 63 collections across all regions (Fig. 1). Age differences between regions >5 ka would in almost all instances significantly lower the correlation coefficient. However, the exact form of the trend line cannot be predicted *a priori* because of uncertainties in the magnitude of the glacial-age temperature depression for each region. Consequently, the A/I–MAT relation is a necessary, but not sufficient condition to confirm that all collections of burnt *Genyornis* eggshell are of the same age.

### Temporal distribution of burnt *Dromaius* and *Genyornis* eggshell

**Figure 5:**
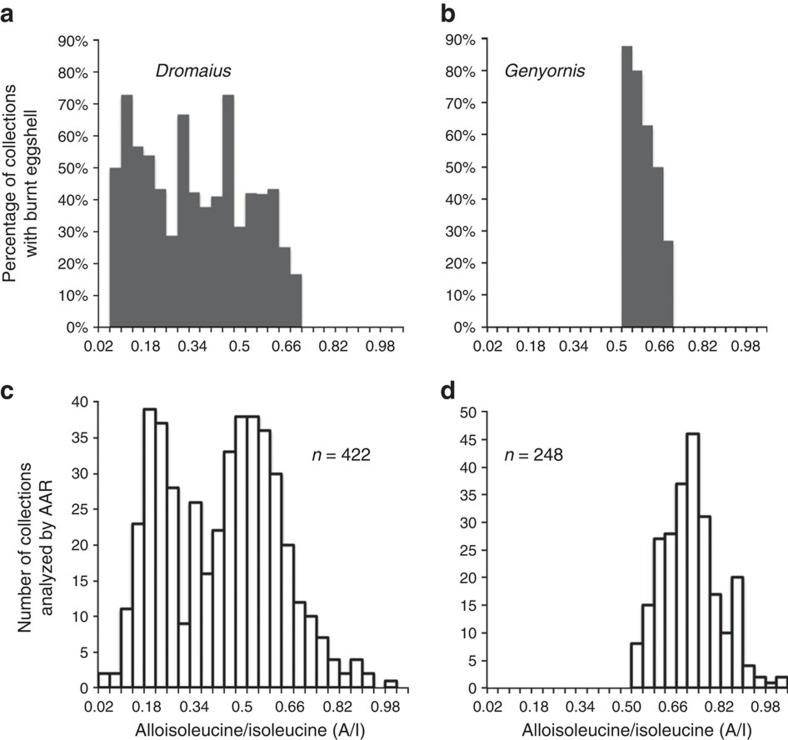
Temporal distribution of burnt *Dromaius* and *Genyornis* eggshell in collections from Western Australia. The lowest A/I in each collection is used to characterize the age of all fragments, to exclude fragments with accelerated racemization resulting from heating by cooking fires. (**a**) Percentage of all WA *Dromaius* collections that contain burnt eggshell, binned in 0.04 A/I units. (**b**) Percentage of all WA *Genyornis* collections that contain burnt eggshell binned in 0.04 A/I units. (**c**) Total number of WA *Dromaius* collections characterized by AAR and binned in 0.04 A/I units. (**d**) Total number of WA *Genyornis* collections characterized by AAR and binned in 0.04 A/I units. The percentage of collections containing burnt *Genyornis* eggshell (**b**) increases as the number of *Genyornis* collections (**d**) decreases, reflecting the difficulty of eliminating localized heating effects on the measured A/I. The likely extinction window for *Genyornis* is 0.54±0.04 A/I units. Isoleucine racemizes 16% faster in *Dromaius* eggshell relative to *Genyornis*^28^; the first appearance of burnt eggshell fragments is at the same age for both taxa. *X* axis scales are identical in all panels.

To further test whether burnt eggshell is diagnostic of human predation, we utilize the temporal distribution of collections containing burnt *Genyornis* and *Dromaius* eggshell derived from AAR analyses. If human predation is the sole cause of variably burnt *Genyornis* eggshell fragments, then similarly burnt *Dromaius* eggshell should first appear in the record ∼50 ka, occur continuously to the present, but never occur before human arrival. In our WA collections, where burnt eggshell of both taxa are most common, burnt *Dromaius* eggshell first appear in sites with AAR indicative of 50±5 ka, indistinguishable within stated uncertainties to the dates associated with burnt *Genyornis* eggshell, remain frequent in collections through to near-modern time, but are not present in any collection >55 ka in the four WA regions (Fig. 5a) or in any of the other regions. Similarly, none of the *Genyornis* eggshell collections from WA that predate 50±5 ka contain burnt fragments (Fig. 5b); the increasing percentage of burnt *Genyornis* eggshell in the lowest three A/I bins is consistent with human predation leading to *Genyornis* extinction.

## Discussion

We found no *in situ* hearths or *in situ* stone artefacts directly associated with burnt *Genyornis* eggshell, or with similar-age burnt *Dromaius* eggshell, and only rarely in association with pre-Holocene, post-45 ka burnt *Dromaius* eggshell. This is expected because the alkaline dune sediments that preserve eggshell carbonate also degrade charcoal^29^, and transient cooking fires in a sandy substrate leave no baked clays. Few regions provided *Genyornis* eggshell in stratigraphic sections where their association with artefacts could be securely evaluated; most samples were collected from the floors of deflation hollows. Lithic artefacts (commonly) and hearthstones (occasionally) are found in deflation hollows among surface scatters of burnt *Genyornis* eggshell, but because both occur as deflationary lags, temporal association cannot be demonstrated. However, the presence of hearthstones confirms that fire-using humans were in the same landscape, despite the lack of preserved hearths. At Garnpung (Fig. 1), one of the few sites with burnt *Genyornis* exposed in a stratigraphic section, an *in situ* hearthstone stratigraphically below the horizon containing burnt *Genyornis* eggshell (Supplementary Fig. 4) demonstrates a temporal overlap with humans.

The time interval during which *Genyornis* became extinct (50±5 ka)^8^ coincides with the interval when humans were consuming its eggs (53.9 to 43.4 ka), suggesting that predation contributed to the bird's extinction. This is also the same interval when the dietary intake of *Dromaius* underwent a dramatic reduction in the proportion of C4 grasses, with C4 dietary elements remaining reduced through to the present^30^. An explanation for the sudden shift in *Dromaius* diet remains obscure, but the loss of palatable C4 grasses across the arid zone would have placed additional stress on *Genyornis* survival, as their diet always included some C4 grass elements^30^.

Our interpretation of burnt eggshell places greatest reliance on the chronological patterns. Burnt *Genyornis* eggshell only occur in a narrow temporal window between 54 and 43 ka defined by dated collections containing burnt eggshell across the arid zone, and this window coincides with both the extinction of the species^8^ and the dispersal of people across Australia^6^,^7^. Furthermore, the oldest similarly burnt *Dromaius* eggshell are dated to the same time window, persist to contemporary time, but are absent from our extensive collections dated between 55 ka and >100 ka. The range of burn patterns found in clusters of *Genyornis* eggshell is most consistent with humans scattering eggshell fragments of consumed eggs in and around transient cooking fires, and the strong thermal gradients required to explain the observed burn patterns are incompatible with a wildfire cause. OSL dating limits the timing of *Genyornis* egg predation to no older than 53.9 ka, or younger than 43.4 ka, and likely no younger than 47 ka, based on the ^14^C dates (see also Supplementary Data 3 for details). AAR correlations expand to 63 the number of dated collections from ten regions across the continent with burnt *Genyornis* eggshell. Our data provide compelling evidence that humans not only dispersed rapidly across Australia's well-watered landscapes, but also deep into its arid interior at or before ∼47 ka, preying on at least one element of the megafauna, the eggs of the giant bird, *Genyornis newtoni.* We hypothesize that human predation on *Genyornis* eggs likely contributed to the birds' extinction, with the harvesting of their eggs decreasing *Genyornis* reproductive success. Predation, combined with widespread changes in ecosystem composition throughout its range^30^, very likely caused *Genyornis* extinction by ∼47 ka.

## Methods

### Amino-acid analyses

Eggshell are mechanically cleaned by grinding to remove surface impurities and the outer portion of each eggshell. For *Dromaius* eggshell, which has a tripartite structure, the outer two layers are mechanically removed. After grinding, an additional 33% of the remaining eggshell mass is removed by the stoichiometric addition of 2 N HCl, with the reaction driven to completion *in vacuo*. Cold 7 N HCl (spiked with the non-protein amino-acid norleucine (6.25 × 10^−5^ mol l^−1^, to enable absolute concentrations for each amino acid to be determined) is added to a ∼15 mg subsample of the cleaned fragment in proportion to sample mass. The resultant solution is flushed with N_2_, sealed and heated at 110 °C for 22 h to hydrolyse protein residues. Amino acids are separated by automated high-performance liquid chromatography (Agilent 1100/1200 HPLC) utilizing ion-exchange and post-column derivitization with o-phthalaldehyde. Amino-acid concentrations are derived by comparing peak areas of individual amino acids to the area of the norleucine spike. The proportion of D-alloisoleucine to L-isoleucine (A/I) is based on peak–height ratios. All samples are analysed at least twice. A natural standard (ILC-G^31^) is analysed daily to monitor instrumental precision.

### Optically stimulated luminescence

The dated sites are well bleached, finely stratified aeolian deposits lacking any visible evidence of post-depositional bioturbation or other mixing. Sediment samples for OSL dating were collected by driving a 7 × 25 cm^2^ stainless steel tube horizontally into a cleaned vertical face stratigraphically related to levels with burnt *Genyornis* eggshell. Bulk sediment from 30 cm diameter around the sampled site was collected for U, Th and K concentrations, which were measured by neutron activation analysis and delayed neutron activation (NAA/DNA); K was calculated from measurements of K_2_O by X-ray fluorescence. Radioisotope activities for U, Th and K were also measured by high-resolution gamma spectrometry and subsequently converted to concentrations; these data confirmed secular equilibrium in the U and Th decay chains. Cosmic ray dose rates were calculated using the data of ref. 32, making allowance for site altitude, geomagnetic latitude and time-averaged thickness of sediment overburden. Alpha-particle irradiation from radioisotopes within the etched quartz grains was assumed to be 10% of the external activity, and the efficiency with which alpha-particle irradiation induced OSL (*a*) was assumed to be 0.05±0.02. Long-term water content was estimated from measured values and reconstructions of the landscape history and topographic position, with uncertainties sufficient to accommodate all likely possibilities.

In the laboratory, 90–125 μm quartz grains were isolated from each sediment sample under low-intensity red and orange light. OSL measurements were performed on ∼5–6 mg of etched quartz attached by silicone oil to the central 7 mm diameter of each of 128 stainless steel discs. The OSL signal was measured on an Elsec Type 9010 automated reader with 500±80 nm stimulation, and ultraviolet emissions detected by an EMI 9235QA photomultiplier tube optically filtered by one UG 11 and one U-340 filter and P determined by the ‘Australian slide' using a linear plus single saturating exponential fit (scale factor=1.00). More complete details are in ref. 33. The advantage of multiple-grain OSL over single-grain OSL for these stratified low-dose-rate sediments is that the use of many grains effectively eliminates small-scale dose heterogeneity by averaging out the slight grain-to-grain differences in beta dose.

### Radiocarbon dating

After mechanical cleaning to remove all secondary carbonate, eggshell mass was further reduced by stoichiometric addition of 2 N HCl to remove 75% of the remaining eggshell mass. CO_2_ was evolved from the cleaned eggshell fragment, purified and converted to graphite at the Laboratory for AMS Radiocarbon Preparation and Research, University of Colorado Boulder, and measured at the W.M. Keck Carbon Cycle Accelerator Mass Spectrometry Laboratory, University of California Irvine. Conventional radiocarbon dates were calibrated using Calib 7.1 and SHcal13 (ref. 34).

## Additional information

**How to cite this article:** Miller, G. *et al.* Human predation contributed to the extinction of the Australian megafaunal bird *Genyornis newtoni* ∼47 ka. *Nat. Commun.* 7:10496 doi: 10.1038/ncomms10496 (2016).

## Supplementary Material

Supplementary Information Supplementary Figures 1-5 and Supplementary References

Supplementary Data 1 Field ID, Region (Fig. 1), and location of all collections containing burnt *Genyornis* eggshell.

Supplementary Data 2 Extent of isoleucine epimerization (A/I) for each collection containing burnt *Genyornis* eggshell that has been analyzed from the ten regions shown in Fig. 1. In most collections some fragments were heated sufficiently to accelerate racemization, but not hot enough to blacken the eggshell. To minimize the bias introduced by unrepresentative A/I we limited the averaging to samples with at least two fragments that difference by no than 0.02 A/I units above the lowest A/I in the collection. For a few collections we used the lowest A/I if that was the only analysis within 1 σ of the region's mean A/I. Collections for which there were no samples within 0.02 A/I units of the lowest, or within 1 σ of the region's mean are considered "unresolved" and were not utilized for any statistical analyses ("1" in the "unresolved" column). Shading is used to aid in differentiating different collections within a region. Samples that were so heated that all amino acids had decomposed are given an A/I of 99999.

Supplementary Data 3 Details of sites from which multiple dating methods have been applied. *Youngest of 9 OSL dates from the Genyornis-bearing unit at this site (supplementary ref. 3)

## Figures and Tables

**Table 1:** Primary geochronological data for sites with burnt *Genyornis* eggshell.

**Region**	**Site**	**MAT (**°C**)**	**OSL±1σ (ka)**	^**14**^**C (conv)±1σ**	**Cal BP** **−1σ**	**Cal BP +1σ**	**Avg A/I±1σ (n)**
*Geochronology for sites with burnt* *Genyornis* *eggshell*							
B	Cardabia Sliver	24.3		44,110±1,430	45,974	48,753	0.67±0.01 (4)
W	Fabulous	23.4	53.6±4.4	40,060±860	43,008	44,410	0.66±0.01 (3)
W	Z-Blowout	23.4	47.9±2.3	40,850±960	43,504	45,145	0.57±0.01 (3)
W	Upper 12-Mile	23.4	45.3±3.5	45,360±1,660	47,445	>50,000	0.67±0.01 (4)
GN	Small Slot	23.2		>44,350	>48,040		0.58±0.02 (10)
Q	Borrow Pit	23.0		>41,800	>45,380		0.57±0.01 (4)
Q	Sunset-1	23.0	47.2±3.5	48,790±3,640	47,444	>50,000	0.58±0.01 (6)
E	Williams Point	21.1	50.8±3.3	44,630±1,520	46,517	49,283	0.51±0.02 (26)
PB	Wood Point	17.9	55.0±5.0	42,400±1,760	44,094	47,419	0.37±0.01 (19)
A	Outer Arumpo	17.1		39,830±840	42,864	44,220	0.34±0.01 (6)
GA	Garnpung	17.1	43.1±1.6	42,010±1,100	44,317	46,310	0.34±0.01 (9)
WL	Wallaroo	17.0		42,630±1,190	44,752	47,035	0.35±0.01 (3)
D	Perry Sandhills	16.8		42,600±1,900	44,630	47,132	0.34±0.01 (7)
							
*Geochronology for sites with* *Genyornis* *lacking burnt fragments, but with low A/I for their MAT*							
B	Ningaloo	24.3		45,060±930	47,438	49,427	0.67±0.03 (3)
W	11-Mile	23.4	47.3±4.9				0.68±0.01 (8)
Q	Sunset-2	23.0	52.1±4.8				0.67±0.03 (11)
E	Williams Point	21.0		42,830±880	45,194	46,874	0.51±0.02 (26)
E	Williams Point	21.0		45,440±1,210	47,776	49,877	0.51±0.02 (26)
E	Hunt Peninsula	21.0		42,930±720	45,410	46,792	0.49±0.02 (3)
E	North Harbour	21.0	59.1±3.9	40,800±580	43,773	44,863	0.52±0.02 (4)
S	Geny Heaven	19.1		42,250±360	43,329	45,871	0.37±0.01 (3)
S	Coopers Dune	19.1	44.7±2.1				0.39±0.01 (25)
S	Mystery Is. Sp1	19.1	55.5±2.3	43,770±630	46,240	47,664	0.46±0.03 (16)
S	Mystery Is. Sp2	19.1	47.3±1.7				0.51±0.08 (5)
S	Flinders North	19.1	55.0±3.0				0.43±0.01 (4)
D	Nialia Lake	17.1		46,600±2,600	>50,000		0.35±0.01 (4)
D	Tandou; Buffy Sand	17.1		46,000±790	48,768	>50,000	0.43±0.01 (4)
D	Tandou; Double Red	17.1		43,230±590	45,790	47,005	0.39±0.01 (2)
D	Kangaroo Lake	17.1		50,330±1,300	>49,000		0.47±0.01 (5)
D	Menindee	17.1	51.0±4.6				0.56±0.04 (12)
D	Lake Victoria	16.8		>44,400	>47,637		0.41±0.01 (4)
	Mean OSL age		51.5±4.9				
							
*Geochronology for sites with* *Genyornis* *known to be >70 ka*							
B	George's Dune	24.3		42,390±110			0.88±0.02 6)
W	Lower 12-Mile	23.4	89±8	45,040±140			1.00±0.01 (3)

Two sites known by AAR (B, W) and/or OSL (W) to be more than 70 ka, returned ^14^C ages similar to ages on burnt *Genyornis* eggshell that are known to be much younger (OSL), documenting the limitations of ^14^C dating of eggshell carbonate that is more than 40 ka. A/I, D-alloisoleucine to L-isoleucine; Avg, average; MAT, mean annual temperature; OSL, optically stimulated luminescence; Cal BP, calibrated years before present; A: Arumpo Station, NSW; B: Bullara, Ningaloo, and Cardabia stations, WA; D: Lower Darling River, NSW; E: Lake Eyre, SA; GA: Garnpung Station, NSW; GN: Sites on and around Gnaraloo Station, WA; PB: Port Broughton, SA; Q: Sites on and around Quobba Station, WA; WL: Wallaroo, SA; W: Sites on and around Warroora Station, WA.

Sites are ordered in each panel by their current MAT. ^14^C dates calibrated with Calib 7.1 and SHcal13.
