# Supplementary material for: Human predation contributed to the extinction of the Australian megafaunal bird Genyornis newtoni ∼47 ka
Source: Nat Commun. 2016 Jan 29;7:10496. doi: 10.1038/ncomms10496 (PMC4740177; doi:10.1038/ncomms10496)
Supplement: Supplementary Information — Supplementary Figures 1-5 and Supplementary References [file ncomms10496-s1.pdf]

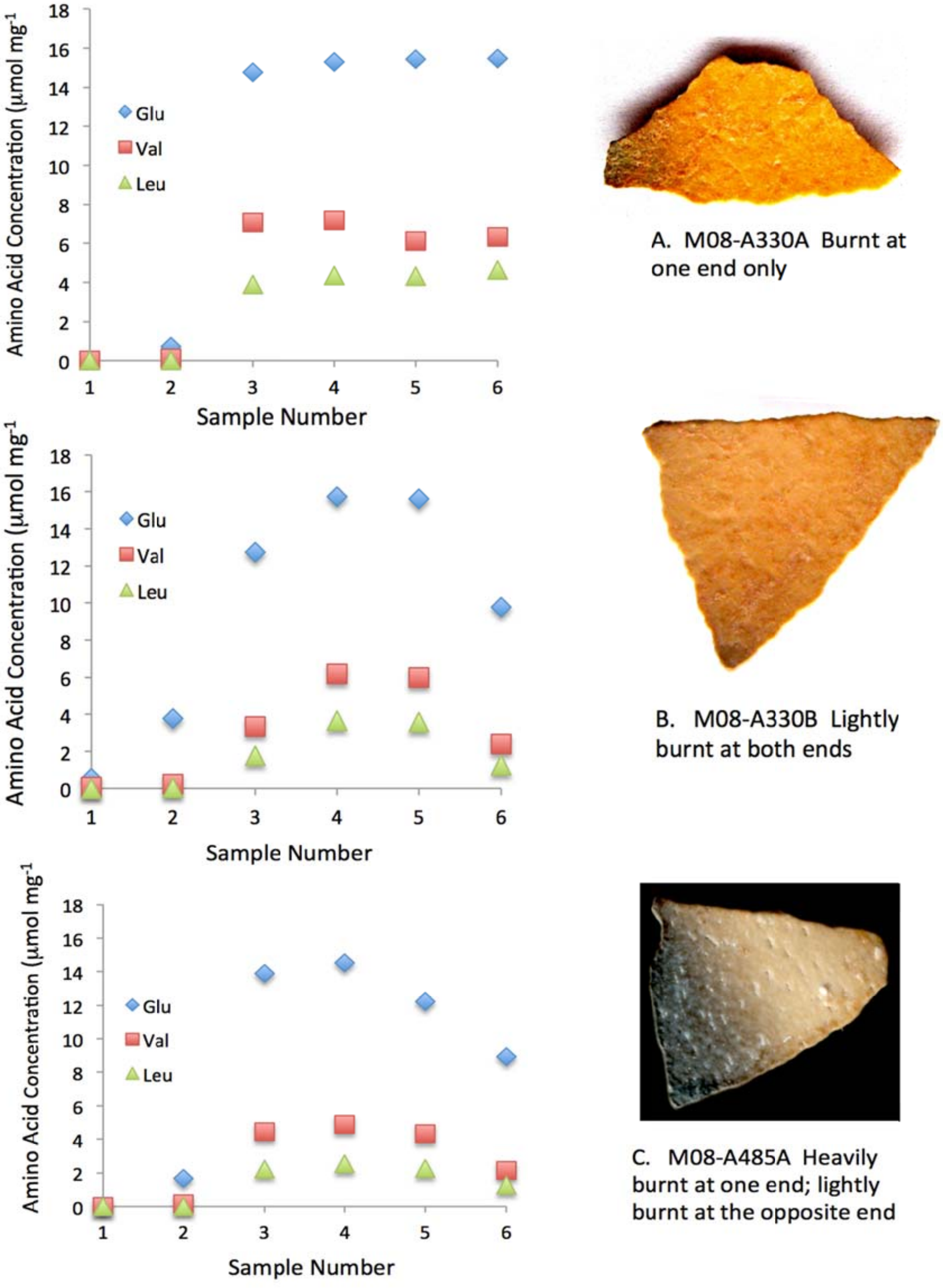

Supplementary Figure 1. Amino acid transects through three variably burnt *Genyornis* eggshell showing complete decomposition of intracrystalline amino acids at blackened ends, variably less decomposition farther from charred regions, approaching unburnt amino acid concentrations farthest from charring. The stable amino acids Glu = Glutamic Acid, Val = Valine, Leu = Leucine are shown, less stable amino acids were more depleted.

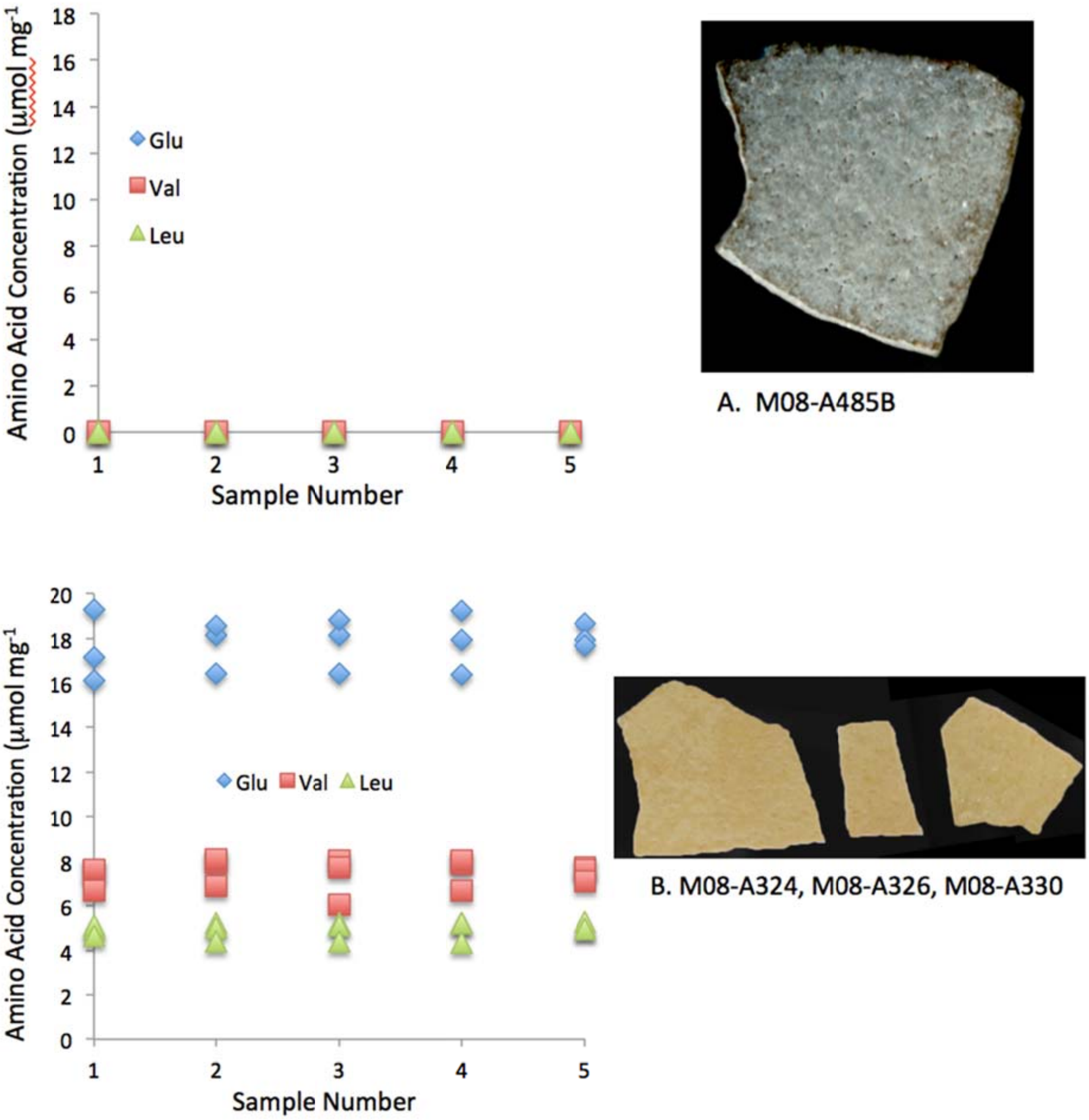

Supplementary Figure 2. Concentrations of the stable amino acids glutamic acid (Glu), valine (Val) and Leucine (Leu) in burnt and unburnt *Genyornis* eggshell. A) Amino acid transects through a fully blackened *Genyornis* eggshell from region "Q" (Fig. 1) showing complete decomposition of intracrystalline amino acids at all locations, indicative of heating in excess of 500 °C throughout the fragment, whereas other fragments from the same collection have A/I consistent with an age of 47.5 ± 2.5 ka (Supplemental Data 2). B) Amino acid concentrations through unblackened eggshell from three different collections in region "W" (Fig. 1), exhibiting no signs of burning. M08-A330 collection includes some variably burnt eggshell, presumably from the same egg as the analyzed fragment.

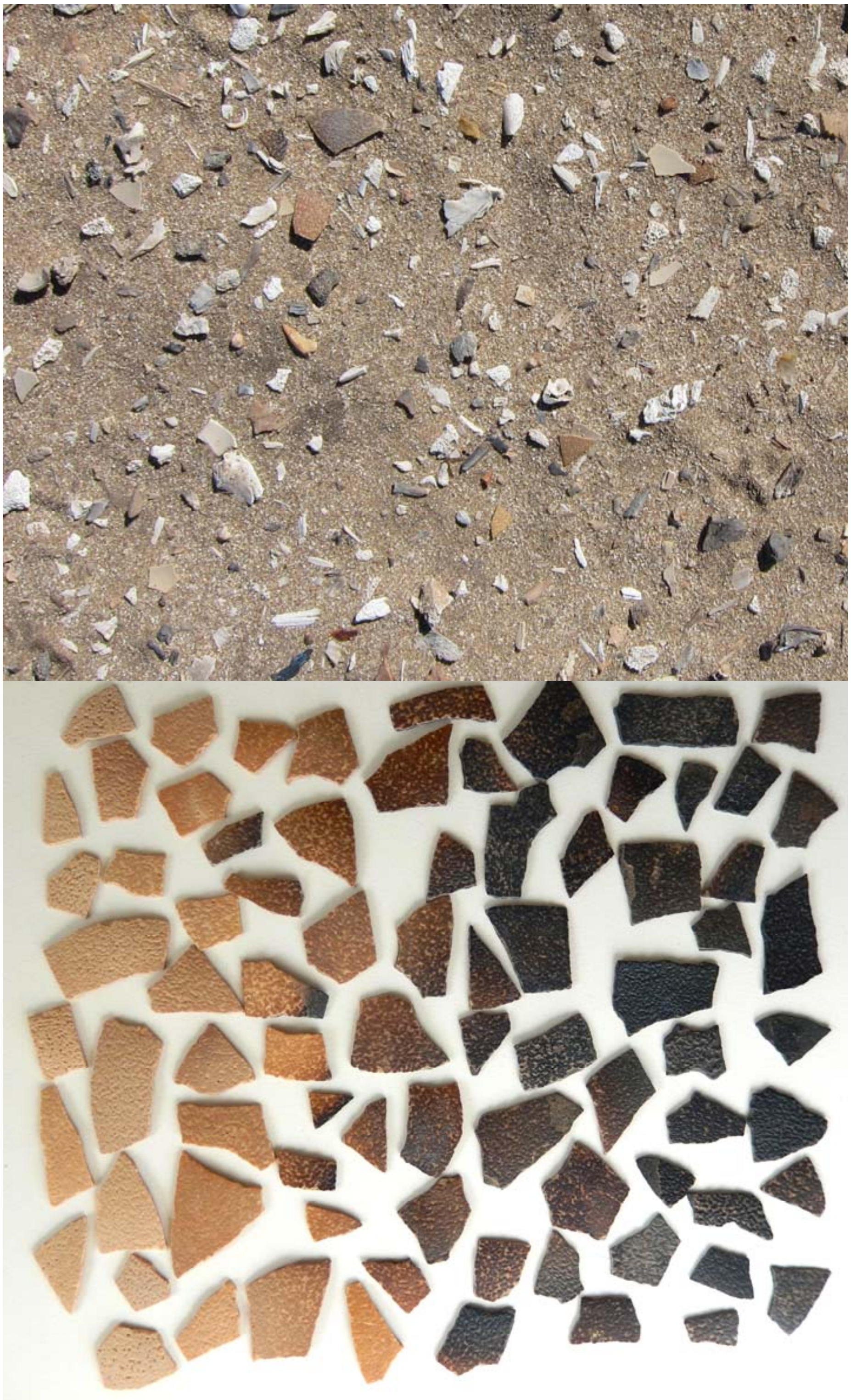

Supplementary Figure 3. Variably burnt *Dromaius* (emu) eggshell the display similar burning patterns to those found on *Genyornis* eggshell from the same region. A) Recent deflation of a late Holocene kitchen midden in region "W" (Fig. 1) containing variably burnt *Dromaius* eggshell and abundant marine shellfish and occasional vertebrate bone, both of which also exhibit burning. B) Fragments of a single late Holocene *Dromaius* egg from the south coast of Western Australia that include variable burning patterns.

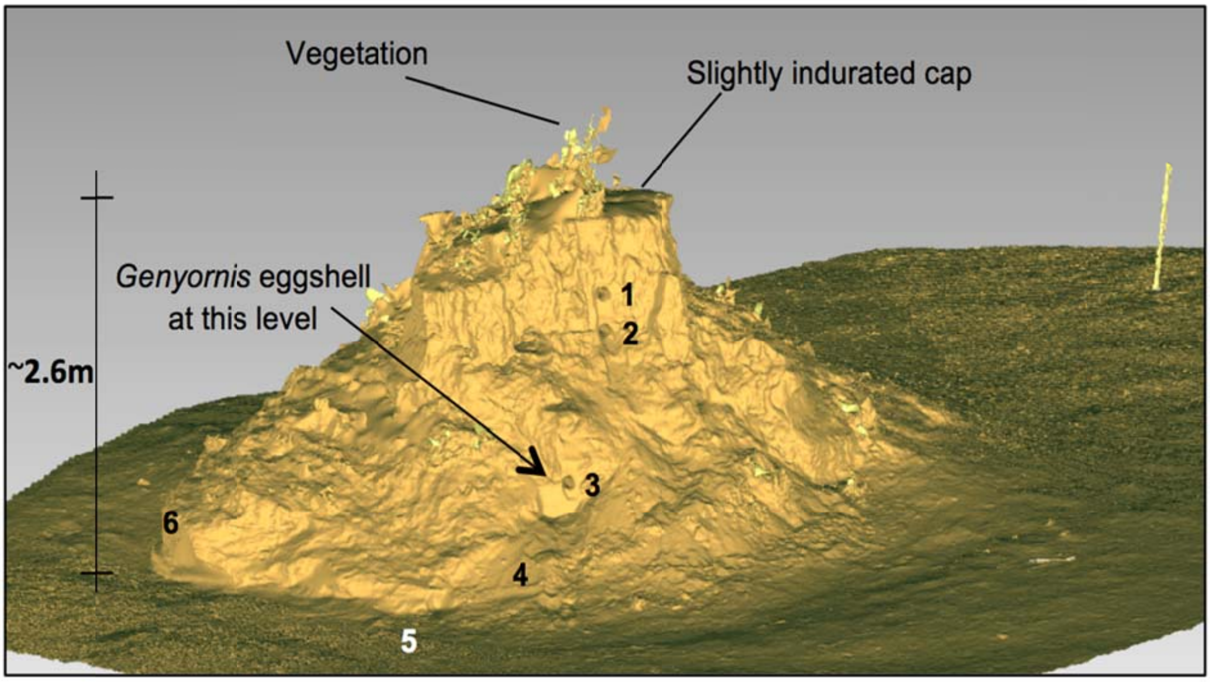

Genyornis AAR (A:I)

Lab ID	A:I
AAL-13994B	0.329
AAL-9944E	0.330
AAL-13994D	0.336
AAL-13994C	0.347
AAL-13994A	0.348
AAL-13873E	0.349
AAL-9944A	0.354
AAL-13873F	0.359
AAL-9944C	0.373
AAL-9944B	0.374
AAL-10766A	0.982
AAL-10766C	1.041
AAL-10766B	1.044
AAL-10765A	burnt
AAL-10765B	burnt
AAL-10765C	burnt

Genyornis 14C age

Lab ID	Conv. ^{14}C age
NSRL-26734	42,010 ± 1100 BP

OSL dates

Lab ID	OSL age ±1σ
Adl2098	23.1 +/- 0.6 ka
Adl2099	23.5 +/- 0.9 ka
Adl2100	43.1 +/- 1.6 ka
Adl 2101	58.7 +/- 2.4 ka

3D Scan of residual in site GG-16, Willandra Lakes Region World Heritage Area, NSW.

1. GG16-1 (0.62m) **23.1± 0.6**
2. GG16-2 (0.94m) **23.5± 0.9**
3. GG16-3 (1.80m) **43.1± 1.6**
4. GG16-4 (2.60m) **58.7± 2.4**
5. Floor of blow out with surface scatter of *Genyornis* egg shell and stone artefacts
6. Hearthstone in situ

Supplementary Fig. 4 Garnpung GG-16 3D scan of aeolian residual with OSL dates located. *In situ* burnt and unburnt *Genyornis* eggshell excavated at "3"; hearthstone located at "6" is stratigraphically below the *Genyornis* collection, based on tracing stratified sediment in the field. AAR in 16 excavated *Genyornis* reflect the common range of A:I found in variably burnt collections, with some fragments having elevated A:I. The lowest six A:I are used to define the mean A:I for this site. 3D scan by E. Beckett and D. Williams (unpub.); OSL dates by N. Spooner (unpub.).

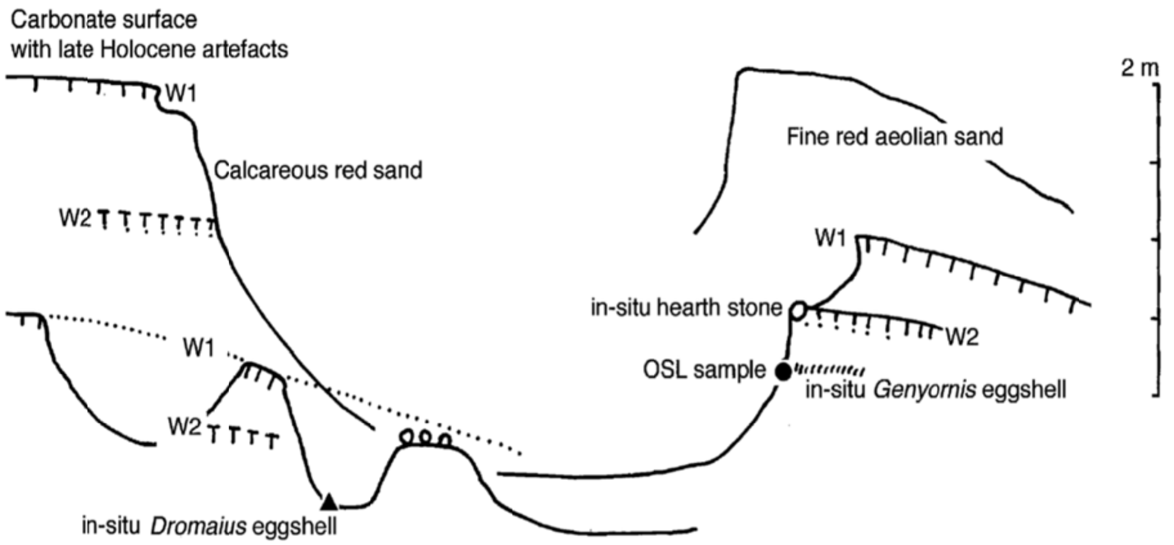

Lab-ID	A/I		Lab ID	A/I
AAL-6931B	0.355		AAL-6931A	0.382
AAL-13883D	0.359		AAL-13996B	0.382
AAL-13992D	0.360		AAL-13992B	0.385
AAL-6931C	0.361		AAL-13883A	0.386
AAL-6931F	0.361		AAL-13883C	0.388
AAL-13992A	0.361		AAL-13996D	0.404
AAL-13995D	0.362		AAL-13996A	0.406
AAL-13992C	0.366		AAL-13883B	0.411
AAL-6860D	0.370		AAL-13995B	0.419
AAL-6931E	0.371		AAL-13883E	0.422
AAL-6860A	0.372		AAL-13995A	0.436
AAL-6860C	0.372		AAL-13995C	0.440
AAL-6931D	0.375		AAL-13996C	0.459
AAL-6860B	0.380			

Genyornis ^{14}C age

Lab ID	Conv ^{14}C date
AA-10238	42,400 ± 1760 BP

OSL date on quartz enclosing *Genyornis* eggshell

Lab-ID	OSL Age
*	55,000 ± 5,000 yr

Supplementary Figure 5. Wood Point site at region "PB" (Fig. 1) with a section sketch [1] and details of OSL[2], ^{14}C and AAR (both from this paper) results. Vertical scale as shown, horizontal scale relative. The lowest 13 A/I (0.355 to 0.375) were used to calculate a mean value for the site; higher A/I are presumed to have been heated; burnt eggshell is common at this site (Fig. 2). Eggshell collections by D.L.G. Williams, G. & M. Miller, and M. Smith.

Supplementary References

1 Bird, M. I. *et al.* Radiocarbon dating of organic- and carbonate-carbon in Genyornis and Dromaius eggshell using stepped combustion and stepped acidification. *Quaternary Science Reviews* **22**, 1805-1181 (2003).

2 Roberts, R. G. *et al.* New ages for the last Australian Megafauna: Continent-wide extinction about 46,000 years ago. *Science* **292**, 1888-1892 (2001).

3 Cupper, M. L. & Duncan, J. Last glacial megafaunal death assemblage and early human occupation at Lake Menindee, southeastern Australia. *Quaternary Research* **66**, 332-341 (2006).
